# Impact of Whole Cereal–Pulse Flours on the Functionality and Antioxidant Properties of Gluten-Free Extruded Flours

**DOI:** 10.3390/foods14203515

**Published:** 2025-10-15

**Authors:** Franz J. Chuqui-Paulino, Davy W. Hidalgo Chávez, José L. Ramírez Ascheri, Caroline Grassi Mellinger, Jhony W. Vargas-Solorzano, Carlos W. Piler Carvalho

**Affiliations:** 1 Postgraduate Program in Food Science and Technology, Federal Rural University of Rio de Janeiro, Seropédica, Rio de Janeiro 23890-000, Brazil; franzchuqui@gmail.com (F.J.C.-P.); davyhw76@gmail.com (D.W.H.C.); 2 Embrapa Agroindústria de Alimentos, Avenida das Américas 29501, Guaratiba, Rio de Janeiro 23020-470, Brazilvargasjw@gmail.com (J.W.V.-S.)

**Keywords:** extrusion conditions, functional properties, physical properties, bioactive compounds, plant-based products

## Abstract

Extruded whole flours from blends of cereals and pulses have great potential to be key ingredients in the development of more innovative gluten-free products, both from a technological and nutritional perspective. The objective of this work was to obtain pre-cooked flours from four formulations based on blends of whole cereals (PR: parboiled brown rice; PM: pearl millet) and pulses (CP: chickpea; CB: common bean). CB was fixed at 10%, and the other components (PR-PM-CP) were set at 60-15-15 (F1), 15-60-15 (F2), 15-15-60 (F3), and 30-30-30 (F4), which were extruded at two combined conditions of feed moisture and screw speed: mild E1 (30% and 300 rpm) and severe E2 (18% and 600 rpm). The temperature profile was kept constant from 25 to 130 °C (from feed to output). The protein, dietary fiber, and ash contents in the raw formulations varied from 11.2 to 17.4%, 9.8 to 15.0%, and 2.2 to 3.3%, respectively, according to the low or high pulse content in the blend. As more mechanical energy was delivered to the raw formulations (W·h/kg, 63.7 for E1 and 179.4 for E2), the extruded particles had increased water absorption (g/g) from 1.7 to 4.5 (E1) or 3.8 (E2), increased water solubility due to E2 from 10.9 to 20.9%, and decreased oil absorption (g/g) from 1.5 to 0.9 (E1 and E2). The peak viscosity (PV, cP) was noticeable only in the raw formulation F2 (355), which decreased 10.3% due to E1. In the other formulations, PV appeared due to E1 in F1 (528), F3 (420), and F4 (371), while it disappeared due to E2 in all formulations. However, at the E2 condition, they did show cold viscosity in the initial stage (222 to 394 cP). The final viscosity (FV, cP) decreased from 795 to 390 (E1) or 123 (E2). In F2, the contents of phenolic compounds (285 µg GAE/g) and ABTS^+^ (13.2 μmol TE/g) were more than twice that in the other formulations, and their respective degradations were low due to E1 (4.2 and 12%) and high due to E2 (16 and 17%). Extrusion cooking did not cause significant changes in the luminosity (81) and redness (0.9) of particles, while yellowness increased from 15.7 to 18.2 (E1) or 18.7 (E2). Based on these findings, it is concluded that both extrusion conditions improved the technological and functional properties. Regarding the formulations, F2 stood out for being rich in antioxidant capacity, which poorly degraded under the conditions studied. Further work is needed to contribute to understanding the optimization of formulas and processes that would improve the nutritional, sensorial, and functional properties while still preserving the bioactive value of the final products.

## 1. Introduction

The market for gluten-free products has experienced significant growth in recent years, driven by the risen prevalence of celiac disease, gluten sensitivity, and a broad consumer interest in healthier dietary options [1]. The need for healthier and more nutritious diets has prompted the development of new products using functional ingredients, primarily hydrocolloids and proteins derived from plant sources. However, the extraction of hydrocolloid and protein requires substantial energy and often involves chemical reactants that produce effluents, both of which are known contributors to environmental issues. A more sustainable approach to producing gluten-free and functional ingredients could be achieved through physical processing methods, such as extrusion cooking technology, particularly when using whole flours from cereals and pulses as feed materials, which would also enhance the nutritional aspects (mostly dietary fiber, minerals, and bioactive compounds) [2]. Extrusion cooking inactivates the enzymatic system present in raw whole flours, providing stability to the final product. According to the processing conditions, many of the bioactive compounds in whole flours may be retained in the final product [2]. It is well established that the seed coat of dried grains provides dietary fibers, phenolics, and flavonoids, all of which are beneficial to human health [3]. Pulses are important sources of protein and other healthful components such as dietary fibers, resistant starch (RS), minerals, and phenolics [4]. By combining whole flours from cereals and pulses, the protein content is increased, the amino acid profile is complemented, and balance is achieved. Depending on the proportion of primary biopolymers (starch and protein) in the feed material and operating conditions of the extruder, starches and proteins may undergo modifications that would favor their functional properties. The operating conditions of extrusion cooking include the injection of water into the feed material, the input of thermal energy along the barrel, and the input of mechanical energy via the rotational speed of the screws [5].

Extrusion cooking is a versatile and efficient process used in the production of various food products, including breakfast cereals, snacks, extruded pastas, and pre-cooked flours for varied food applications, which can be adjusted to the dynamic changes in food habits and lifestyles of the people [6]. Using this technology to process whole-grain cereals and pulse flours can be appealing for the development of innovative food products with enhanced nutritional and functional characteristics [7,8]. As an example of using the extrusion technology, mixtures of maize and lentil flours were used to produce a precooked gluten-free flour by extrusion at 130 °C. The authors emphasized the effects on starch conversion, protein denaturation, and reduction in antinutritional compounds, which were considered key aspects for improving digestibility and broadening application in instant food products. With this mixture, enhanced antioxidant activity, reduced glycemic index, and improved techno-functional behavior of the resulting precooked gluten-free flour were observed [1]. However, this work did not study the effect of varied extrusion processes, which would limit the effect in a range of low and highly modified pre-cooked flour. In any case, extrusion cooking offers a significant advantage to the food industry by enabling the transformation of cereal-pulse flours into nutrient-dense, low-fat food products. Moreover, recent developments have highlighted the use of extruded flours as innovative, sustainable ingredients in food formulations, serving functions such as hydrocolloids and fat replacers and enabling the creation of gluten-free, phenolic-enriched, low-glycemic, and functional food products [8].

The functionality of powdered ingredients, such as extruded flours, depends on the behavior of their constituent particles and biopolymers in the environment in which they are dispersed. This includes factors such as water absorption, water solubility, oil absorption, bulk density, and paste viscosity [5,9,10]. These properties are influenced by extrusion parameters, including temperature, screw speed, feed moisture content, and feed composition [9]. In extrusion cooking technology, process parameters vary based on the raw ingredients and the requirements of the final product. The quality of the extruded product is determined by the combination of all process parameters and blend formulation, requiring further study. Therefore, this study evaluated the impact of two extrusion cooking conditions on four whole flour blends based on cereals (parboiled brown rice and pearl millet) and pulses (chickpea and common bean). The aim was to assess changes in bioactive compounds and functional properties of extruded flours, with the goal of identifying ingredients with potential applications in gluten-free products.

## 2. Materials and Methods

### 2.1. Plant Materials and Blend Formulation

Pearl millet (PM) was kindly donated by Atto Sementes (Rondonópolis, Brazil), while parboiled brown rice (PR), carioca bean (CB), and chickpea (CP) were donated by Granfino Alimentos (Nova Iguaçu, Brazil). Particularly, pearl millet grains were processed on a grain cleaner (Clipper Separation Technologies, Bluffton, SC, USA). All grains were subsequently ground into fine flours using an LM3100 hammer mill (Perten Instruments, Huddinge, Sweden) equipped with a 0.8 mm opening.

The proportions (in %) of parboiled brown rice (PR), pearl millet (PM), chickpea (CP), and carioca bean (CB) flours in four raw formulations (PR/PM/CP/CB) were designated as follows: F1: 60/15/15/10; F2: 15/60/15/10; F3: 15/15/60/10; and F4: 30/30/30/10. The CB percent was fixed in all formulations, so the addition of a superior proportion causes bean flavor, as reported by Mariscal-Moreno, R. M., C. Chuck-Hernández, J. D. Figueroa-Cárdenas and S. O. Serna-Saldivar [11], who found that by adding 10% of beans in bread making, changes in flavor were not sensorily perceived.

### 2.2. Extrusion Cooking

The extrusion process was carried out in a co-rotating twin-screw extruder, Evolum HT25 (Clextral Inc., Firminy, France), with a length-to-diameter ratio of 40:1. This extruder featured ten temperature zones that were kept constant at temperatures of 25, 25, 50, 80, 110, 110, 120, 120, 130, and 130 °C, from the feed to the output. The extruder was also equipped with a two-blade cutter, GR2008 (Clextral Inc., Firminy, France), used to cut the extrudates into small pieces to facilitate the milling for the production of pre-cooked flours.

The blended whole meal flours were fed into the feeding zone using a twin-screw, loss-in-weight gravimetric feeder model GRMD15 (Schenck Process, Darmstadt, Germany) at a constant rate of 10 kg/h. The Schenck Process Easy Serve software version 31.19 (Schenck Process, Darmstadt, Germany) monitored the process. Deionized water was injected between the first and second modular zones of the extruder barrel using a plunger-metering pump, Super K PP 6.35 (DKM Clextral Inc., Firminy, France), for moisture adjustment. Two extrusion conditions were considered, denominated E1:30% of feed moisture (FM) and 300 rpm of screw speed (SS), and E2:18% of feed moisture and 600 rpm of screw speed. The extrudates were dried at 60 ± 5 °C overnight in a forced-air drier. Then, they were sealed in plastic bags and stored at 7 °C for subsequent analysis.

### 2.3. Bulk Density

The bulk density (BD) of both raw and extruded flour formulations (Supplementary Materials) was determined using the method described by Vargas-Solórzano, J. W., J. L. R. Ascheri, C. W. P. Carvalho, C. Y. Takeiti and M. C. Galdeano [12]. The flour sample, without being compacted, was freely poured into a graduated cylinder until it reached a volume of 50 cm^3^. In order to create a particle bed, the cylinder was placed under the outlet of a single-screw volumetric vertical feeder (Brabender, Duisburg, Germany), which dispensed 50 g of flour at a rate of 4 kg/h. This feeding rate was determined by interpolating curves that relate the feeder’s rotational speed (in rpm) to the mass of particles collected over 1 min.

### 2.4. Distribution of Particle Size

The particle size distribution was determined using isopropyl alcohol as a dispersant in S3500 laser equipment (Microtrac Inc., Montgomeryville, PA, USA) using the light scattering technique [13] on both raw and extruded blend flours. In addition, particle-size distribution curves were generated on the cumulative percentage of the sample. These curves identified the diameters of particles (μm) corresponding to the 10% (D_10_), 50% (D_50_), and 90% (D_90_) finer percentiles. The characterization of the particle size distribution was determined by the Span value, calculated using Equation (1):

(1) $$Span=\left(\frac{{D_{90}-D}_{10}}{D_{50}}\right)\times 100$$

### 2.5. Sample Preparation for Hydration Analysis

The extrudates were ground in a LM3100 hammer-mill (Perten Instruments AB, Huddinge, Sweden) equipped with a 0.8 mm sieve aperture. To reduce the influence of particle size, as recommended by Becker, A., S. E. Hill and J. R. Mitchell [14], the particles were sieved, and those retained between 106 and 212 μm openings were used for water absorption (WAI) and solubility indices (WSI), oil absorption index (OAI), and paste viscosity measurements.

#### 2.5.1. Water Absorption Index (WAI) and Water Solubility Index (WSI)

The procedure followed the methodology described by Vargas-Solórzano, J. W., C. W. P. Carvalho, C. Y. Takeiti, J. L. R. Ascheri and V. A. V. Queiroz [15]. WSI and WAI analyses were conducted in triplicate and were calculated using Equations (2) and (3).

(2) $$WSI\left(\%\right)=\frac{gwatersolublematter}{gdrysample}\times 100$$

(3) $$WAI\left(g\;gel/g\right)=\frac{gwaterabsorbed}{gdrysample\times \left(1-solublefraction\right)}$$

#### 2.5.2. Oil Absorption Index (OAI)

The procedure was conducted in accordance with Ahn, H. J., J. H. Kim and P. K. W. Ng [16]. For the determination of OAI, 0.5 g of the flour sample was weighed into a 50 mL pre-weighed centrifuge tube and thoroughly mixed with 5 mL of vegetable oil (100% soybean oil). The flour-oil mixture was centrifuged (2000× *g* for 20 min). Immediately after centrifugation, the supernatant was carefully removed, and the tube was weighed. The OAI was calculated using Equation (4).

(4) $$OAI=\frac{goilabsorbed}{gdrysample\times \left(1-solublefraction\right)}$$

#### 2.5.3. Paste Viscosity

A Rapid Visco Analyzer series 4 RVA (Newport Scientific Pty Ltd., Warriewood, Australia) was used to measure the paste viscosity of both raw and extruded blend samples, following the methodology reported by Carvalho, C. W. P., C. Y. Takeiti, C. I. Onwulata and L. O. Pordesimo [17]. A 3 g sample adjusted to 14% moisture (wb) was used. The recorded pasting property readings were pasting temperature (PT, cP), cold viscosity at 25 °C (CV, cP), peak viscosity (PV, cP), trough viscosity or holding strength (HS, cP), breakdown viscosity (BV = PV − TV, cP), final viscosity (FV, cP), and setback viscosity (SV = FV − TV, cP).

### 2.6. Bioactive Compounds

To determine the bioactive compounds, it was necessary to produce the extracts of the raw materials, along with the extruded formulations. The extracts were obtained using the procedure described by Rufino et al. [17], which was employed as follows: the sample flours were weighed (g) in centrifuge tubes and extracted sequentially with 40 mL of methanol/water (50:50, *v*/*v*) at room temperature for 1 h, and the effect of light was avoided, then centrifuged at 2000× *g* for 15 min, and the supernatant was recovered. Then, 40 mL of acetone/water (70:30, *v*/*v*) was added to the residue at room temperature, extracted for 60 min, and centrifuged. Methanol and acetone extracts were combined, made up to 100 mL with distilled water, and used to determine antioxidant capacity and extractable polyphenol content.

#### 2.6.1. Total Phenolic Compounds (TPC)

The total phenolic compounds (TPC) were determined using the Folin–Ciocalteu method described by Singleton and Rossi (1965), modified by Georgé, S., P. Brat, P. Alter and M. J. Amiot [18]. An aliquot of Folin–Ciocalteu reagent (1.25 mL at 10%) was added to 250 mL of the extract and reacted for 2 min at room temperature. Then, 1 mL of sodium carbonate (7.5% *w*/*v*) was added, taken to a water bath at 50 °C for 15 min, and cooled in an ice bath. The absorbance was measured at 760 nm in a UV–Vis spectrophotometer (Shimadzu 1800, Kyoto, Japan), and the blank was prepared by replacing the sample with distilled water. Gallic acid (5–130 mg/L) was used as a standard, and a calibration curve was constructed. Measurements were performed in triplicate, and the results were expressed in equivalent micrograms of gallic acid/g of dry weight sample (µg GAE/g). In addition, a standard gallic acid curve as a reference at 760 nm was used (Y = 0.0095X + 0.0064, R^2^ = 0.9995).

#### 2.6.2. Antioxidant Capacity (ABTS^+^ Radical Scavenging)

The antioxidant capacity was measured according to the method described by Re, R., N. Pellegrini, A. Proteggente, A. Pannala, M. Yang and C. Rice-Evans [19] by radical scavenging of ABTS (2,2′-azino-bis (3-ethylbenzothiazoline-6-sulfonic acid)). From the sample extract, a 30 μL was homogenized with 3 mL of diluted ABTS^+^ solution (7 mM ABTS^+^ 140 mM potassium persulfate + 95% ethanol). The absorbance was read 6 min from the moment of the addition of the radical. Simultaneously, 30 μL of ethanol was used as a blank for the analysis. Additionally, a standard curve of Trolox at five concentrations (Y = 0.0003X − 0.044, R^2^ = 0.9987) was used.

#### 2.6.3. Antioxidant Capacity (DPPH Radical)

The antioxidant capacity was also determined by the method proposed by Brand-Williams, W., M. E. Cuvelier and C. Berset [20], based on the capture of the DPPH (2,2-diphenyl-1-picryl-hydrazyl) radical. A 25 μL aliquot of each extract dilution was transferred into a dark environment and homogenized with 975 μL of DPPH radical. Methyl alcohol served as a blank. Absorbance readings were taken after 30 min at 515 nm using a UV-1800 spectrophotometer (Shimadzu Corporation, Kyoto, Japan). The antioxidant capacity was expressed as Trolox concentration per gram of sample, calculated using the equation y = 0.0006x − 0.0402 (R^2^ = 0.9968).

### 2.7. Emulsion Activity and Emulsion Stability Index

The emulsifying activity index of all samples was determined according to de Paiva Gouvêa, L., R. Caldeira, T. de Lima Azevedo, M. C. Galdeano, I. Felberg, J. R. Lima and C. Grassi Mellinger [21] for all samples.

### 2.8. Instrumental Color

Color parameters were quantified using the reflectance method as per Bernardo, C. O., J. L. R. Ascheri, D. W. H. Chávez and C. W. P. Carvalho [22], using the CIELAB scale with a Chroma meter (Konica Minolta, Tokyo, Japan). The parameters measured included L* (luminosity, where 0 = black and 100 = white); a* (ranging from −80 to 0 for green, and 0 to +100 for red); and b* (ranging from −100 to 0 for blue and 0 to +70 for yellow).

### 2.9. Statistical Analysis and Multivariate Data Analysis

The raw flour blends data were analyzed using a completely randomized design with one-way ANOVA, followed by Tukey’s test in instances where differences were detected.

A 4 × 2 factorial design was employed to investigate the effect of extrusion. The first factor was the blend formulation (F1, F2, F3, F4), while the second factor was the extrusion conditions, which varied in terms of moisture (%) and screw speed (rpm) with levels E1: 30/300 and E2: 18/600. The data from this segment of the experiment were assessed using a two-way ANOVA followed by Tukey’s test, where significant differences were detected. A significance level of 5% was established for all analyses. Finally, a correlation analysis was conducted to identify potential relationships between variables, considering only statistically significant correlation coefficients (r) with *p*-values ≤ 0.05.

## 3. Results and Discussion

### 3.1. Proximate Composition of the Raw Formulations

Table 1 displays the proximate analysis of the raw flour formulations. As the proportion of whole cereal grains increased in formulations (F1, F2, and F4), there was a significant increase in carbohydrate values (*p* ≤ 0.05) compared to F3, which contained a higher legume content (70%). In contrast, F3 showed the highest protein content (15.7%), followed by F4 (12.36%), which contained 40% legume. In terms of lipid content, F3, F4, and F2 exhibited similar and higher values than F1, which consisted of a larger percentage of parboiled brown rice. Additionally, F3 had the highest content of dietary fiber and ash, followed by F4 and F2 with similar values, while F1 presented the lowest values for these components.

### 3.2. Specific Mechanical Energy (SME)

As expected, E1 exhibited lower SME, ranging from 56.81 to 73.37 W·h/kg, compared to E2, which ranged from 151.64 to 208.6 W·h/kg (Table 2). This can be attributed to the high levels of feed moisture in E1, which reduced the shear forces associated with the lower screw speed [23,24]. On the other hand, the combination of low feed moisture levels and high screw speed in E2 resulted in an increase in SME, as reported by Ali, S., B. Singh and S. Sharma [25]. Furthermore, higher SME values were observed in formulations with increased cereal content (F1, F2, and F4) under both extrusion conditions. Specifically, F1 and F2, formulated with 70% cereal, corresponded to carbohydrate contents of 72.4% and 66.86%, respectively, while F4, with low cereal content (60%), had 65.93% of carbohydrates. This trend suggests that formulations with higher carbohydrates signify high starch content, therefore leading to increased SME, hence molecular breakdown directly affecting the rheological properties of the melt [17,26]. Similarly, a higher proportion of pulse (70%) in F3 may have contributed to the reduction in SME in both conditions, due to its elevated protein and lipid content in the formulation. A previous study found that the addition of fish protein (5–20 g/100 g) led to a decrease in SME [5].

### 3.3. Bulk Density (BD)

The bulk density (BD) of the raw flours ranged from 0.39 to 0.53 g/cm^3^ (*p* ≤ 0.05). F1 had the highest BD, followed by F2 and F4, which were similar in both, and F3 had the lowest value (Table 2). The variation in BD was associated with the particle composition, primarily due to differences in the starch fraction, as reported by Anberbir, S. M., N. Satheesh, A. A. Abera, M. G. Kassa, M. W. Tenagashaw, D. T. Asres, A. T. Tiruneh, T. A. Habtu, J. A. Sadik, T. A. Wudineh, et al. [27] in their study, blends composed of teff, pearl millet, and buckwheat. The elevated protein and dietary fiber content in F3 reduced its starch fraction, thereby decreasing its BD. This is in contrast to F1, which had the highest rice composition, indicating a high starch content. Similarly, Lagnika, C., P. A. Houssou, V. Dansou, A. B. Hotegni, A. M. O. Amoussa, F. Y. Kpotouhedo, S. A. Doko and L. Lagnika [28] reported that BD can be influenced by the particle size and the structure of starch polymers.

The BD of the extruded flour formulations varied between 0.55 and 0.59 for E1 and between 0.51 and 0.58 for E2, respectively (*p* ≤ 0.05). Compared to the raw formulations, an increase in BD was observed under both extrusion conditions (Table 1). However, a slight decrease was noted for E2 compared to E1. This suggests that with an increase in feed moisture (FM) and low screw speed SS (E1), BD increased, whereas with low FM and increasing SS, BD decreased. This aligns with results obtained during the production of weaning food based on corn-bean [29] and corn-chickpea [25]. Higher moisture content reduces internal friction within the matrix, therefore decreasing the drag force and preventing high temperature and pressure at the exit [30]. This, in turn, reduced melt viscosity, causing a laminar flow of the melt and restricting the expansion, which increased the BD of the extrudates [31,32]. An increase in BD was also observed due to the composition of the feed material used in the extrusion process, particularly the presence of fiber, which undergoes a minimum reduction in size while remaining firm, helping in the compaction of the extruded material [32]. In the work of Mongi, R., C. Ruhembe and K. Urembo [33], an increase in soy flour in millet-soybean formulations resulted in a significant reduction in BD. This was attributed to moisture being the primary variable contributing to the increase in density values, followed by the proportion of each grain and their interaction used in the formulation [34]. On the other hand, an increase in screw speed led to a reduction in the density of the extruded material due to the structural breakdown of macromolecules under high shear conditions [32].

### 3.4. Particle Size Distribution (PSD)

**Figure 1:**
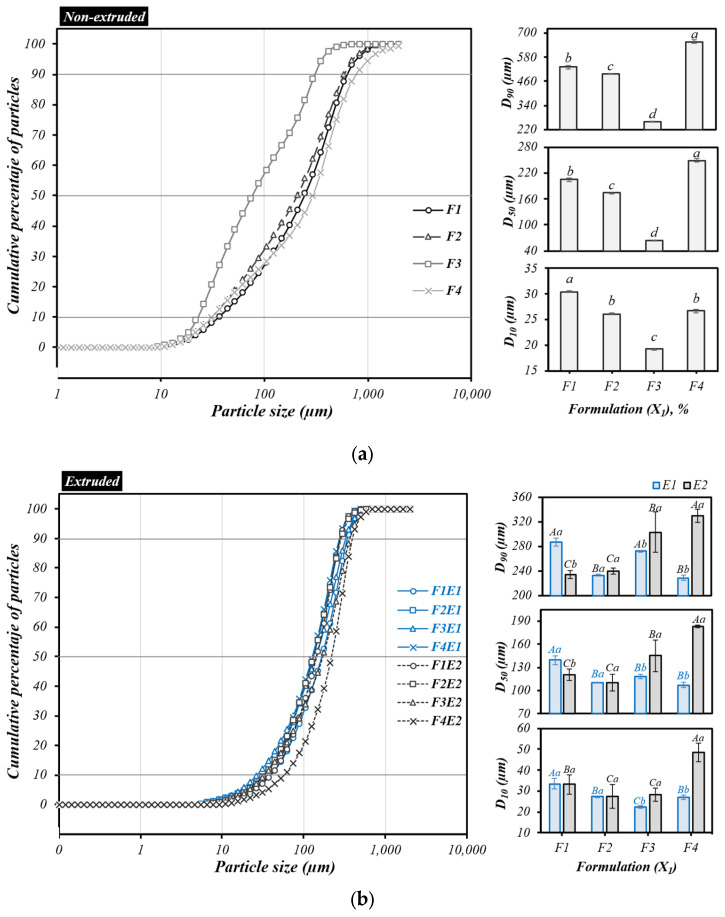
Cumulative particle-size distribution of whole-grain flour formulations: raw (**a**) and extruded (**b**). For non-extrusion flours, different lower-case letters at the right side in the bar chart indicate differences (*p* ≤ 0.05) among blend formulations. For extrusion samples, different uppercase letters at the right side of the bar chart in the same bar color indicate differences (*p* ≤ 0.05) among the formulations inside the same extrusion condition. The same lowercase letters on the right side of the bar chart in the different bar colors indicate similarities (*p* > 0.05) between extrusion conditions for each blend formulation. E1, E2 are extrusion conditions (%moisture/rpm); F1–F4 (%/%/%/%): blends of percentages of parboiled brown rice (PR), pearl millet (PM), chickpea (CP), and carioca beans (CB).

The particle size distribution of raw formulations showed significant differences (*p* ≤ 0.05). PSD plays an important role in functional, physicochemical, adsorption, and hydration [35]. D_10_ ranged from 19.22 to 30.4 µm, D_50_ ranged from 64.21 to 249.27 µm, and D_90_ ranged from 259 to 656.9 µm. Formulations with the highest cereal content (F1, F2, and F4) had the largest fractions in the PSD, as shown in the cumulative curve (Figure 1a). Conversely, inclusion of chickpea flour in F3 resulted in smaller fractions in the PSD, as evidenced by the curve’s leftward shift, indicating a sample with a smaller particle size. This could be attributed to the soft structure of the chickpea cotyledon, which disintegrated into smaller particles during milling.

On the other hand, the PSD of extruded flours revealed that, under the influence of E1, D_10_ ranged from 22.32 to 33.5 µm, D_50_ from 106.83 to 139.47 µm, and D_90_ from 229.02 to 287.03 µm. Under the influence of E2, D_10_ ranged from 27.47 to 48.63 µm, D_50_ from 109.8 to 182.92 µm, and D_90_ from 234.1 to 329.25 µm (Figure 1b). The results indicated larger sizes in F1 due to the influence of E1 and in F4 due to the influence of E2, as evidenced by the rightward shift in the cumulative percentage curve of particles. In contrast, smaller particle sizes were observed in the other formulations, as indicated by a leftward shift [36]. Furthermore, a variable PSD was observed, with significant differences between each formulation (*p* ≤ 0.05). These differences could be attributed to factors such as hardness, type, processing mode, milling method, or technique [37].

### 3.5. Functional Properties

#### 3.5.1. Water Solubility and Water Absorption Indexes

The water absorption index (WAI) of the raw flour formulations varied from 1.28 to 2.09 g/g (*p* ≤ 0.05). Among raw mixtures (Table 3), F1, which is rich in rice, displayed the highest WAI (2.09 ± 0.19 g/g), followed by F4 (1.79 ± 0.05 g/g). In contrast, F3, which contains a significant proportion of chickpea, showed the lowest WAI (1.28 ± 0.10 g/g). The elevated WAI in F1 can be ascribed to the capacity of parboiled rice to retain water due to its partial gelatinization during the parboiling process. Furthermore, this property is greatly influenced by the particle size and structure of the starch granules [38], which contribute to the overall water absorption capacity. Conversely, the reduction in WAI observed in the F3 formulation could be attributed to increased protein content resulting from the high percentage of chickpea in its composition. This observation aligns with the findings reported by [38], who proposed that an increase in the interactions between the hydrophilic groups of starch and protein, particularly present with the addition of legume flours, hinders the water retention capacity of starch, leading to a decrease in the WAI.

Following the extrusion process, significant differences in WAI (*p* ≤ 0.05) were found under both extrusion conditions (Table 3). Under condition E1 (30% moisture/300 rpm), WAI values varied between 3.85 and 5.39 g/g, with the highest value recorded for F1-E1 (5.39 ± 0.52 g/g). This indicates that blends rich in parboiled rice responded most effectively to this milder thermomechanical treatment, promoting optimal starch conversion without excessive molecular degradation. In contrast, extrusion condition E2 (18% moisture/600 rpm) consistently produced lower WAI values, ranging from 2.92 to 4.26 g/g, with a marked reduction compared to E1 for all formulations. This is likely attributable to the more intense shear in E2, which may have led to molecular structure degradation, thereby reducing water-binding sites and overall water absorption capacity. A decrease in WAI values in E2 compared to E1 was observed across all formulations. The use of high feed moisture and low feed rate in E1 increases the WAI of extrudates [31], inducing a plasticizing effect that reduces shearing, hence starch conversion [39]. Conversely, high screw speeds and low feed moisture intensify shear and friction forces, leading to changes in starch granules through fragmentation, which results in mechanical damage and a reduction in the WAI [40]. Additionally, it was observed that WAI increases with a higher proportion of parboiled brown rice (F1) and pearl millet (F2) in the formulation, as both showed higher carbohydrate content. High WAI values are a good indicator of damaged starch fragments in the final product [39]. On the other hand, an increase in legume content decreases the WAI, as observed in rice- and chickpea-based extrudates [31], as well as with increasing soybean meal in millet-soybean meal formulations [33].

The water solubility index (WSI) of the raw flour formulations varied significantly, ranging from 7.76 to 16.82% (Table 2). It was observed that the WSI began to increase as the proportion of whole grain decreased (from 75 to 30%) and that of pulses increased (from 25 to 70%) in the raw flour formulations. This increase could be attributed to the presence of soluble components. Raw F3 had the highest WSI (16.82%), possibly due to the higher content of chickpea-derived soluble proteins and carbohydrates (soluble fibers). In contrast, the lowest WSI was found in F1 (7.76%), which could be attributed to the low presence of soluble components that are found in F3, rich in chickpeas. The observed variations in value could be due to the WSI serving as an indicator of the degree of molecular degradation and solubilization [27]. This suggests that not only the starch but also water-soluble components like proteins are important for a higher WSI [31]. Furthermore, Sharma, R., S. Sharma, H. A. Makroo and B. N. Dar [30] also argued that protein plays a more significant role in dictating the solubility of starch molecules.

Following extrusion, the WSI values for E1 ranged from 6.78 to 12.5%, while for E2, they ranged from 17.84 to 25.94% (Table 3). A substantial increase in WSI was particularly noticeable under extrusion condition E2. The WSI values under E2 reached up to 25.94% for F1 and 21.92% for F4, indicating a relevant molecular breakdown under high shear and low moisture conditions. In addition, a high feed moisture level coupled with a low screw speed in E1 resulted in lower WSI values. Extrusion cooking at low humidity leads to increased starch fragmentation and promotes the formation of water-soluble molecules [32]. This is because high moisture content results in lower WSI, increased plasticization during extrusion, and reduced friction phenomena, thereby exerting a protective effect on flour constituents [41]. Higher solubility values are generally associated with starch degradation, which increases with screw speed [32,42]. In addition, the composition of feed material affects WSI, with lower values being attributed to reduced starch and increased protein content [9,42]. As observed in millet-soybean formulations, a decrease in WSI with an increase in soybean flour up to 30% implies that the composite flour was primarily composed of starch aggregates and had a relatively high content of soybean lipids and protein [33]. These results corroborate our findings, which indicate that intense extrusion enhances solubility by disrupting starch-protein matrices and exposing soluble fragments.

#### 3.5.2. Oil Absorption Index

The oil absorption index (OAI) of the raw formulations varied between 1.31 and 1.72 g/g (Table 2), with F3 showing the greatest oil absorption capacity. The OAI value of F3 was significantly higher than that of the other blends, suggesting that its elevated chickpea content (60%) contributed to increased fat-binding capacity. This effect is likely due to the higher protein and fiber concentrations in F3 compared to the other formulations, which provide a greater number of nonpolar binding sites capable of interacting with lipids [16,43]. In other words, the water-holding and oil-holding capacities of proteins are influenced by the balance between hydrophilic and hydrophobic amino acids that are on the surface of protein particles. This ratio would affect how proteins interact with water and oil, thereby impacting their functional properties in food systems [44]. The main proteins in chickpea flour are globulins, particularly vicilin and legumin, which are rich in hydrophobic amino acids, with leucine being the most prominent. Hydrophobic proteins exhibit superior cohesive strength with lipids, leading to a high OAI in the flours [45].

The extrusion process consistently reduced the OAI values in all blends, with values decreasing to below 1.05 g/g under both extrusion conditions (Table 3). This reduction is expected, as protein denaturation and starch hydrolysis during extrusion often decrease porosity and the availability of hydrophobic binding sites, thereby limiting oil retention. The process conditions used in the extrusion could have influenced the OAI. For instance, in E1, the use of high moisture and low screw speed is consistent with the findings of Lazou, A. and M. Krokida [46], who reported that an increase in moisture, which reduces the degree of cooking of the extrudates, led to a decrease in OAI. On the other hand, Sobowale, S. S., Y. O. Kewuyemi and A. T. Olayanju [47] found that a decrease in moisture resulted in an increase in OAI, a trend observed in E2.

#### 3.5.3. Emulsifying Properties

The emulsifying capacity (EC) exhibited significant variation among the raw samples (Table 3). The balanced blend, F4, showed the highest EC (19.67 ± 2.08%), while F1 had the lowest (9.97 ± 2.32%). This implies that the elevated legume content (CP and CB) in F3 and F4, attributed to their higher protein content, may have contributed to enhancing the functional emulsification properties. de Paiva Gouvêa, L., R. Caldeira, T. de Lima Azevedo, M. C. Galdeano, I. Felberg, J. R. Lima and C. Grassi Mellinger [21] reported that emulsifying capacity is more closely related to the solubility, surface charge, and molecular structure of the protein, as well as with the interactions between protein-protein, protein-oil, and protein-water, rather than merely the water and oil absorption or retention capacities. Furthermore, Stone, A. K., M. G. Nosworthy, C. Chiremba, J. D. House and M. T. Nickerson [48] noted that legumes tend to produce more emulsions than cereals, mainly due to their protein content and elevated levels of albumin- and globulin-type proteins.

The EC of the extruded flours from all formulations showed varying patterns depending on the extrusion conditions (E1 and E2). E1 demonstrated a generally positive impact on EC, with values reaching up to 17.93% (F1–E1) and 17.9% (F4–E1). This increase might be attributed to protein cleavage induced by the extrusion, potentially exposing hydrophobic residues that favored interfacial activity. In contrast, E2 negatively influenced EC for the majority of the formulations. The exception was F2, which displayed an EC increase compared to E1, while the values for other formulations were drastically reduced, with a minimum of 8.35 in F3. Higher EC values could be ascribed to the composition and proportion of the raw materials in the mixture, in conjunction with the extrusion conditions, which could improve the functionality of the formulations. To achieve good emulsifying properties, high protein content and solubility are necessary, facilitating the migration of protein molecules to the oil-water interface, promoting their binding, and, consequently, increasing the EC [24,48]. Conversely, low EC values indicate that the extrusion process parameters could negatively alter the emulsification capacity. For instance, in soybean meal, extrusion has been found to negatively impact emulsification properties, resulting in significantly lower values compared to unprocessed samples [24]. This post-extrusion reduction in protein solubility is recognized as one of the main factors contributing to the decrease in emulsifying properties in extruded soybean meal [24].

The emulsion stability (ES) of the raw flours showed higher values in F2 (81.05%) and F3 (79.60%), indicating superior emulsion stability compared to F1 (68.60%) and F4 (52.22%). This suggests that formulations with a higher content of pearl millet (F2) and chickpea (F3) may facilitate greater interaction among proteins, fibers, and lipids. These blends exhibited higher fiber content (Table 1), which could have enhanced emulsion activity, as this property strongly depends on the structure of proteins and peptides, as well as their interactions with other compounds such as carbohydrates, fibers, lipids, and water [49]. Additionally, pearl millet has been shown to promote the development of viscous matrices under moderate extrusion conditions due to its starch and dietary fiber content. Oliveira, D. P. L., M. S. Soares Júnior, J. A. C. Bento, I. G. dos Santos and T. A. P. d. C. Ferreira [50] reported that millet-enriched extruded snacks exhibited increased water absorption and expansion, indicating the formation of cohesive structures that may support emulsion stability in complex food systems. On the other hand, higher protein content in F3 contributed to the emulsion stability, the same form that soybean, with its higher protein content, exhibited a significantly higher ES than cereal flours, attributing this to the protein content as the main factor contributing to higher ES [48]. In addition, the PSD of F2 and F3 revealed smaller particle sizes, corroborating the findings of Olakanmi, S. J., D. S. Jayas, J. Paliwal and R. E. Aluko [45]. They demonstrated that flours with smaller particle sizes tend to produce more stable emulsions, potentially due to an increased protein adsorption surface, which is capable of forming smaller oil droplet sizes.

In contrast, after extrusion, the ES showed varied responses, ranging from 51.11 to 76.72% under condition E1 and from 43.15 to 87.06% under condition E2. Under E1, F2 maintained a high ES (76.72%), whereas F1 (51.11%) had the lowest value, possibly due to higher starch gelatinization present in the parboiled brown rice. Under E2 conditions, ES values varied across samples; however, the F3 formulation under E2 conditions achieved an impressive 87.06%, suggesting a synergistic effect between the higher protein content from the chickpea-higher blend and the intense extrusion in enhancing emulsion stability. This could be attributed to the composition and particle size variation in the flours, which affect emulsion stability, in conjunction with the severity of the extrusion conditions. Olakanmi, S. J., D. S. Jayas, J. Paliwal and R. E. Aluko [45] reported that flours with smaller particle sizes produced more stable emulsions due to the larger protein adsorption surface area. Furthermore, the higher protein content in these smaller particle sizes ensured stability of the emulsion interface. In contrast, the reduction in protein solubility following the extrusion process can lead to a reduction in emulsion properties, as observed in extruded soybean [24].

#### 3.5.4. Paste Properties

**Figure 2:**
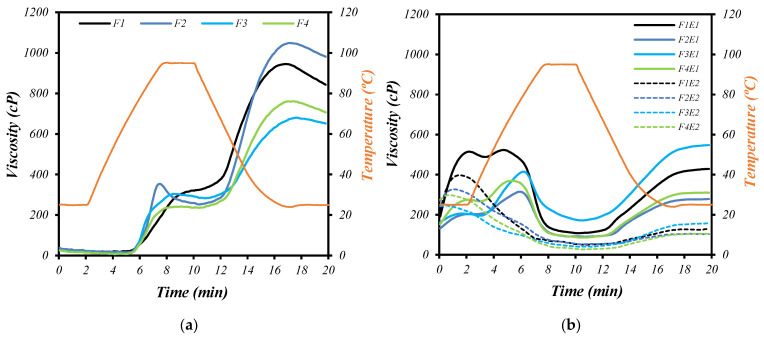
Paste property profile of raw (**a**) and extruded (**b**) formulations of cereals and pulse blends. Extrusion conditions: E1 (30%, 300 rpm) and E2 (18%, 600 rpm) (%moisture/rpm). F1 (60/15/15/10), F2 (15/60/15/10), F3 (15/15/60/10) and F4 (30/30/30/10). The numbers in the brackets are the percentage of each flour, in this order: parboiled brown rice, pearl millet, chickpea, and carioca beans. The orange line indicates the temperature during the analysis.

The pasting behavior of starch-based systems offers insights into their gelatinization, swelling, and retrogradation tendencies, which are key attributes affecting processing and end-product quality. Figure 2 depicts the pasting profiles of raw (a) and extruded (b) flour blends (F1–F4) as determined by a Rapid Visco Analyzer (RVA). The paste viscosity profiles of both the raw and extruded blends are presented in Figure 2.

In relation to the raw blend flours (Figure 2a), no cold viscosity (CV) was observed. F2 (60% pearl millet) exhibited the highest peak viscosity (~1100 cP), suggesting high starch content with good swelling and granule integrity. This aligns with the known gelatinization characteristics of pearl millet starch, which typically exhibits high peak viscosity due to less damaged starch granules and fewer lipid–amylose complexes [51]. F1 (60% parboiled rice) also showed substantial viscosity (~1000 cP), due to its substantial starch content (low in fiber and protein) and the partially gelatinized nature of parboiled rice starch, which readily absorbs water and contributes to high paste thickness even during heating. The presence of pre-gelatinized starch increased the initial viscosity but reduced its stability due to the breakdown of fragmented starch under shear. The parboiling process compromises starch integrity [52]. Conversely, F3 (60% chickpea) and F4 (balanced blend) presented lower peak viscosities (~800–900 cP), indicative of their reduced starch content and higher protein and fiber levels in legume-rich formulations. These components compete for water, impede granule swelling, and disrupt starch paste formation, thereby reducing the overall viscosity. In mixtures of cereal flours with two types of legume flour, Wani, I. A., S. D. Singh, S. Paras and B. S. and Gill [53] found that the PV progressively decreased as the proportion of legume flour in the mixture increased, demonstrating an inverse relationship. The setback viscosities (post-cooling) followed a similar pattern, with F2 exhibiting greater retrogradation potential—a characteristic associated with the presence of amylose-rich starch fractions and low interference from non-starch components. Additionally, it was observed that the FV was higher in F2 than in F1, despite both having the same percentage of cereal in the formulation. A lower FV in F1 may be attributed to the reduced starch content of parboiled brown rice. The heating/cooking process induces an irreversible swelling of the starch in the parboiled rice [52]. In addition, it was also noted that a decrease in the proportion corresponded with a decrease in FV values, a finding consistent with [53], which reported a decrease in final viscosity in cereal-legume mixtures as the substitution of cereal for legumes increased. Muñoz-Pabon, K. S., A. S. Parra-Polanco, D. F. Roa-Acosta, J. L. Hoyos-Concha and J. E. Bravo-Gomez [54] reported that flour composition, including fat, protein, and fiber, could affect starch gelatinization by interacting with the granule, thereby contributing to a reduction in viscosity.

Extrusion cooking markedly altered the pasting behavior of all blends, irrespective of the condition (Figure 2b). Precooked flours showed cold viscosity (CV), with F1 demonstrating higher values than other formulations. Regardless of the formulation, extruded samples displayed reduced peak viscosities, indicative of starch depolymerization and reduced granular integrity due to thermomechanical shear and temperature exposure during extrusion. This effect was more pronounced under the more severe condition E2 (18% moisture/600 rpm). In condition E1 (30% moisture/300 rpm), blends such as F1-E1 and F2-E1 retained moderate pasting ability, with peak viscosities between 600 and 800 cP. The lower shear rate and higher moisture content facilitated partial preservation of granular structure or retrograded starch reactivity, allowing for limited swelling during RVA analysis. Conversely, under E2, samples like F3E2 and F4E2 exhibited a significant reduction in pasting properties (<400 cP), confirming extensive starch degradation. The decreased viscosity under these conditions reflects comprehensive molecular breakdown and formation of thermally stable fragments with limited water-binding capacity [40]. Similarly, the presence of PV was observed in E1 and its absence in E2, the latter being a result of high severity, as PV is dependent on the amount of sheared starch granules [55,56]. Furthermore, the presence of PV in precooked flours by extrusion using relatively high moisture (27%), a speed screw of 200 rpm, and a lower temperature of 70 °C, indicated the presence of partially broken starch granules still capable of water absorption [57]. On the other hand, the absence of PV after extrusion is associated with a significant increase in starch damage, leading to a drop in viscosity [58], as observed by an increase in barrel temperature and screw speed [39].

During the cooling stage, an increase in viscosity was observed, particularly under the E1 condition, likely due to reduced damage to the starch granules compared to E2. Under mild extrusion conditions (E1), not highly sheared starches allowed higher reassociation of amylose-lipid complexes, thus higher retrogradation [15]. However, E2 exhibited a decrease in viscosity at the onset and throughout the cooling stage, possibly due to higher shearing during the extrusion. A slight increase in viscosity was expected after cooling [55]. In another study, extrusion parameters of 27% (FM), 140 °C (BT), and 200 rpm (SS) resulted in less damage to the starch and a reduced tendency to retrograde. This led to a reassociation between amylose molecules, gel formation, and an increase in viscosity during cooling in the RVA [54,58]. Notably, the viscosity curves of E2-treated samples flattened, with minimal breakdown or setback, supporting the hypothesis that thermomechanical treatment induced irreversible structural modifications. It was observed that FV and SV were higher in E1, likely due to the lower severity of extrusion. On the other hand, FV and SV in F3 increased under the influence of the two conditions, surpassing the other formulations. Higher SV values indicate a greater tendency to retrograde [39]. Moreover, the reduction in viscosity in FV and SV caused by the severity of extrusion (E2) demonstrated extensive damage to starch molecules, which lost their retrogradation capacity during cooling [55]. This behavior is often desirable in formulations intended for instant applications, where rapid solubilization and low retrogradation are preferred.

### 3.6. Instrumental Color Measurement

**Figure 3:**
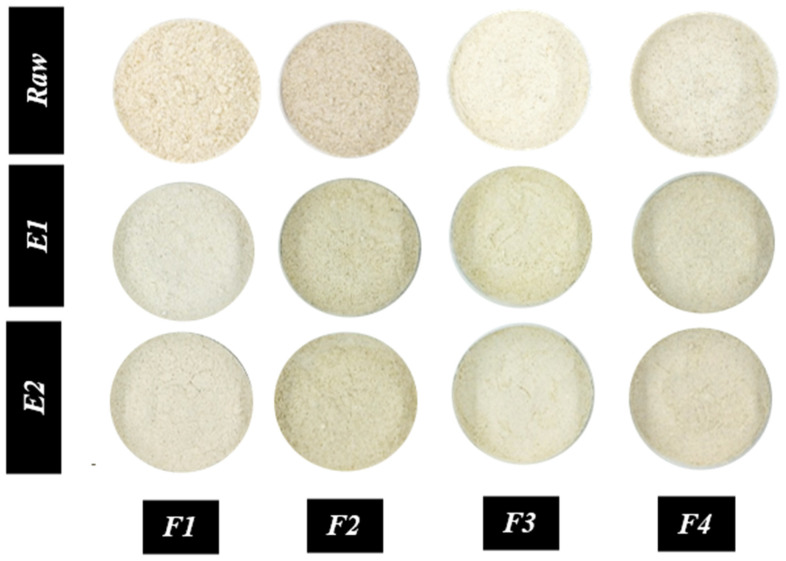
Visual comparison of raw and extruded flours. Extrusion conditions are as follows: E1 (30% moisture, 300 rpm) and E2 (18% moisture, 600 rpm). The flour formulations are F1 (60/15/15/10), F2 (15/60/15/10), F3 (15/15/60/10), and F4 (30/30/30/10). The numbers in parentheses represent the percentage composition of each flour, in the following order: parboiled brown rice, pearl millet, chickpea, and carioca beans.

Color was measured using the CIELAB system parameters (L*, a*, and b*) (Table 4), and significant differences were observed between raw and extruded flour formulations (*p* ≤ 0.05). The visual characteristics of the flours are depicted in Figure 3. In the case of raw flours, a high chickpea content in the formulation resulted in increased brightness (L*) and yellowness (+b*), while a high millet content led to decreased brightness and yellowness but induced an increase in redness (+a*), as illustrated in Figure 3. Koukoumaki, D. I., K. Giannoutsos, P. V. P. Devanthi, P. Karmiris, S. Bourni, A. Monemvasioti, V. Psimouli, D. Sarris and K. Gkatzionis [59] noted that an increase in chickpea flour in the formulation led to an increase in L* values in the production of crackers using pulse flours.

After extrusion, the L* values ranged from 76.85 to 83.41 (with E1) and from 76.55 to 83.01 (with E2). The a* values varied from 0.19 to 1.19 (with E1) and from 0.53 to 1.8 (with E2), while the b* values ranged from 16.44 to 20.09 (with E1) and from 17.93 to 19.71 (with E2). It was observed that extruded flours with a higher proportion of parboiled rice (F1) and chickpea (F3) in their formulation provided greater brightness than other grains. However, L* was not affected by the conditions used, except in the case of F1 (Figure 1b). On the other hand, the a* value, which indicates significant reddening in F2, F1, and F4 due to positive values, and minor reddening in F3, was influenced by high chickpea flour content. The a* values also increased with E2, a trend also observed in the combination of lentil and rice after extrusion by Rico, D., A. B. Cano and A. B. Martín-Diana [1]. The b* value, indicative of yellowness, was higher in F3 and F4 due to the chickpea flour content. These values were not affected by the extrusion conditions, unlike F2 and F1, which exhibited higher values for E2. Alam, M. S., J. Kaur, H. Khaira and K. Gupta [5] found that L* and a* depend on barrel temperature, feed moisture, and screw speed in their study on color changes in the extrusion of corn grits. In conditions of low moisture and high barrel temperature, darkening was enhanced due to Maillard and caramelization reactions, while the effect of high barrel temperature was less significant at higher feed moisture. The screw speed also had a negative impact, as it influenced the residence time [60]. Similarly, Allai, F. M., P. M. Junaid, Z. R. A. A. Azad, K. Gul, B. N. Dar, S. A. Siddiqui and J. Manuel Loenzo [61] observed that an increase in feed moisture and barrel temperature resulted in a reduction in L* and an increase in a* and b* parameters. Pismag, R. Y., M. P. Polo, J. L. Hoyos, J. E. Bravo and D. F. Roa [40] have suggested that enhanced moisture content aids in maintaining L* values and reduces the production of red (a*) and yellow (b*) colors by minimizing thermomechanical effects. Furthermore, an increase in the extruder’s screw speed reduces the residence time, which inhibits the formation of brown colors but may increase the a* and b* parameters [40].

Discussion of functional properties

The functional performance of both raw and extruded blended flours varied significantly based on their formulation and processing conditions. This demonstrates the interactive impact of ingredient composition and thermomechanical treatment on the physical behavior of the blends.

Among the raw blends, F1 (60% parboiled rice) exhibited the highest WAI (2.09 g/g). This correlates with the pre-gelatinized nature of parboiled rice, which promotes water retention. In contrast, F3 (60% chickpea) showed the lowest WAI (1.28 g/g), suggesting reduced starch accessibility due to the legume’s dense matrix. This inverse relationship between chickpea content and WAI was mirrored in the higher OAI (1.72 g/g) for F3, underscoring the superior lipid-binding capacity of chickpea proteins and fibers. WSI values, indicative solubility, and the extent of macromolecular breakdown were particularly high in F3 at 16.82%, further confirming the presence of readily soluble components in chickpeas. In contrast, F1 and F2, which are richer in cereals (PR and PM), displayed lower WSI, associated with more intact starch granules and fewer soluble proteins. Regarding emulsification, F4 (balanced blend) exhibited superior EC at 19.67%, suggesting a synergistic effect of balanced protein and carbohydrate sources. However, F2 and F3 recorded the highest ES values at 81.05% and 79.60%, respectively, likely due to higher protein contents from pearl millet and chickpeas, which contribute to more stable protein films.

Extrusion Condition E1 (30% moisture/300 rpm)

Extrusion under condition E1 significantly increased the WAI in all blends, with the highest value observed in formulation F1 (5.39 g/g). This implies that the matrix dominated by rice was more prone to starch conversion under moderate shear. The high parboiled rice content increased WAI after extrusion, which remained strong across conditions. WSI values exhibited a slight increase for most samples under condition E1, albeit less dramatically than under condition E2, suggesting moderate starch breakdown. Interestingly, despite its high chickpea content, formulation F3 under condition E1 demonstrated only an intermediate WSI (12.50%), possibly due to the stabilizing effect of protein-rich matrices during extrusion. EC improved in all extruded samples, with F4 under E1 and F1 under E1 conditions reaching up to ~18%, indicative of protein unfolding and enhanced interfacial activity. However, ES values exhibited varied trends: formulation F2 under E1 maintained a high ES (76.72%), while F1 under E1 decreased to 51.11%, possibly due to starch-driven aggregation reducing emulsion stability.

Extrusion Condition E2 (18% moisture/600 rpm)

The high shear and low moisture of the E2 condition intensified solubilization, as evidenced by a sharp increase in WSI: F1-E2 (25.94%) and F4–E2 (21.92%). These values correlated negatively with OAI, which decreased in all samples, likely due to the extensive degradation of the starch-protein matrix limiting hydrophobic interactions. WAI values under E2 were generally lower than those in E1, reinforcing the idea that excessive mechanical stress can overly fragment starches, thereby reducing their water retention capacity. For instance, F3–E2 had the lowest WAI (2.92 g/g) among the extruded samples, underscoring the effect of high chickpea content and reduced water absorption under intense processing. The emulsifying capacity remained high in F1, F2, and F4 (~13–17%), although F3–E2 exhibited a great reduction (8.35%), indicating protein denaturation beyond optimal functionality. Notably, F3–E2 displayed the highest ES (87.06%) across all treatments, implying that even denatured proteins from chickpeas can form more rigid interfacial layers under specific processing conditions.

### 3.7. Correlation Analysis of Physical and Functional Properties

**Figure 4:**
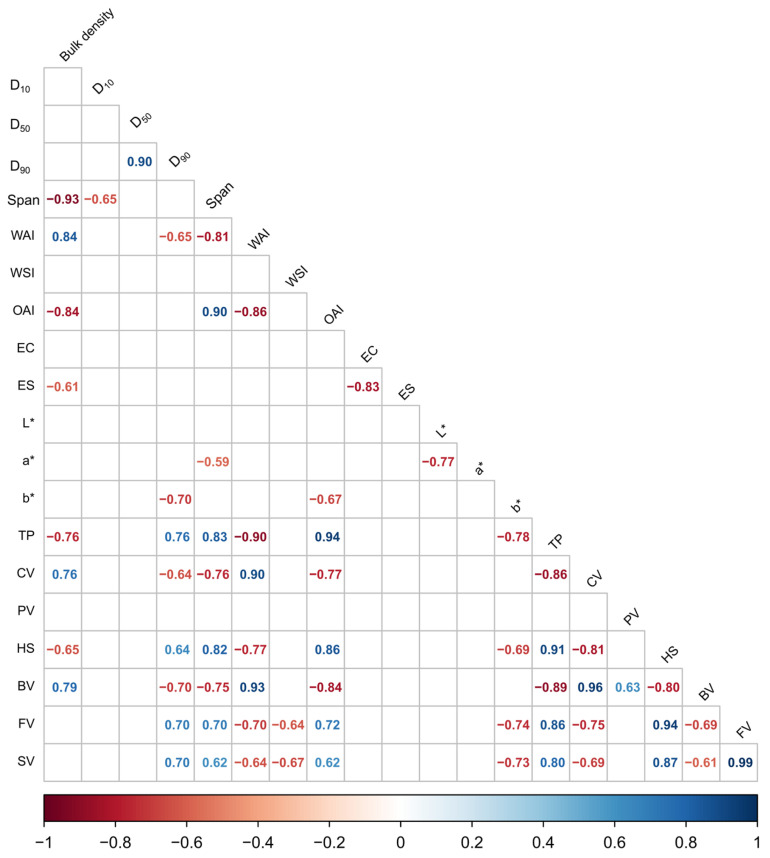
Correlogram of selected responses. D_10_, D_50_, D_90_, and Span are readings of particle size distribution. WAI and WSI are water absorption and solubility, respectively. OAI is the oil absorption index. EC and ES are the emulsification capacity and stability, respectively. L, a*, b* are color parameters. Pasting temperature (PT), cold viscosity at 25 °C (CV), peak viscosity (PV), trough viscosity or holding strength (HS), breakdown viscosity (BV), final viscosity (FV), and setback viscosity (SV) are paste viscosity readings from the RVA curves.

A correlogram was constructed to depict the interplay between certain responses, as illustrated in Figure 4.

In terms of particle size distribution, there were highly positive correlations observed between D_50_ and D_90_ (r = 0.90), indicating an anticipated mutual dependence in particulate systems. The span, a parameter that defines distribution uniformity, was negatively correlated with D_10_ (r = −0.65), suggesting that broader distributions are associated with finer particles.

The water absorption index (WAI) demonstrated a strong positive correlation with cold viscosity (CV) (r = 0.90), indicating that hydration properties are intimately connected to rheological behavior at room temperature. This suggests that the starch matrix, along with other components such as soluble fibers (gums) and hemicelluloses, absorbs water, forming entanglements akin to a 3D network that resists the flow imposed by the paddle of the RVA viscometer. This high correlation implies that severe extrusion cooking results in converted starch structures with a high capacity for water retention, likely leading to an increase in apparent viscosity, particularly observed in the E2 extrusion condition (low moisture content and high screw speed). Unlike mild extrusion conditions, which are expected to cause a certain degree of starch disruption and consequently lead to low cold viscosity, the observed WAI-CV relationship reflects how a higher mechanical shear is associated with a high water absorption index, enabling effective swelling and viscosity at 25 °C.

The pasting viscosity properties were highly interrelated. The final viscosity (FV) was highly correlated with holding strength (HS) (r = 0.94) and initial paste PT (r = 0.86). In contrast, a strong negative correlation was observed between CV and both FV (r = −0.75) and SV (r = −0.69). This inverse relationship would suggest that both extrusion conditions imposed considerable changes in the behavior of starch seen in cold viscosity readings (CV), which are distinct from those values of raw flour contrasting to retrogradation, represented by final viscosity (FV).

### 3.8. Changes in Antioxidant Properties of the Raw Materials, Blends, and Extruded Flours

#### 3.8.1. Total Phenolic Compounds (TPC)

The total phenolic compounds (TPC) are shown in Table 5. Carioca bean (CB) presented the highest TPC value (508.35 mg GAE/100 g), followed by pearl millet (PM) (296.42 mg GAE/100 g). Patil, S. S. and C. Kaur [8] have noted that whole millet grains and pulses are excellent sources of bioactive compounds, such as phenolics and flavonoids, which have been shown to have beneficial health effects.

The TPC of the raw formulations varied from 102.5 to 284.9 mg GAE/100 g (Table 4). The highest TPC content was observed in F2, which contained a larger proportion of pearl millet in its formulation. This formulation was selected as a reference due to its superior TPC to assess the impact of the extrusion process. The extrusion condition presented a minimal reduction under E1 (273 mg GAE/100 g) compared to E2 (238 mg GAE/100 g). In other words, the parameters of E1, characterized by higher moisture content and lower screw speed, may have prevented extensive degradation of the phenolic compounds, in contrast to the parameters of E2, which had lower moisture content and higher mechanical shearing. Extrusion technology influences the bioavailability of phenolic compounds via structural modification, thermal effects, and mechanical shear, and can degrade some phenolics. This could account for the moderate TPC values in extruded flours [62]. Altan, A., K. L. McCarthy and M. Maskan [63] noted that the elevated temperature used in extrusion can modify the molecular structure of phenolic compounds by reducing their chemical reactivity or extraction, and, consequently, causing a loss of their antioxidant properties.

#### 3.8.2. Antioxidant Capacity (ABTS^+^ and DPPH Radical)

The antioxidant capacity of the whole grains, as well as the raw and selected extruded formulations, is shown in Table 5. It was observed that the highest antioxidant capacity for the ABTS^+^ radical was found in the carioca bean (18.43 μmol TE/g), followed by pearl millet (6.98 μmol TE/g). The contribution of these grains to the antioxidant capacity of the raw flour blends was proportional to their inclusion levels in the formulations. Formulation F2 (13.20 μmol TE/g) presented a reasonable value, which decreased by 13.6% when processed with E1 (11.62 μmol TE/g) and by 20.8% with E2 (10.92 μmol TE/g). This formulation was selected for the determination of antioxidant capacity by the DPPH radical due to its higher value compared to other formulations. The raw formulation displayed 9.94 μmol TE/g, which reduced to 5.76 (with E1) and 5.29 (with E2) μmol TE/g after extrusion. Extrusion conditions led to a decrease in antioxidant properties, with reductions of 12% and 16% in ABTS^+^ activity, and more pronounced declines of 42% and 47% in DPPH activity for the E1 and E2 conditions, respectively. Similar results were reported by Martín-Diana, A. B., I. J. Jiménez-Pulido, I. Aguiló-Aguayo, M. Abadías, J. Pérez-Jiménez and D. Rico [62], following the extrusion process for both radicals (ABTS^+^ and DPPH). The decrease in antioxidant properties was influenced by the parameters of the extrusion process, such as feed moisture, temperature [64], and shear forces [62]. Altan, A., K. L. McCarthy and M. Maskan [63] noted that the drawback of natural antioxidants is that their low resistance to high temperatures (above 80 °C) diminishes their antioxidant properties. Similarly, Patil, S. S. and C. Kaur [8] reported that a study conducted on brown rice showed a decrease in total phenolic content and antioxidant activity, with an increase in extrusion temperature.

## 4. Conclusions

This study highlights how the interplay between whole-grain flour composition and extrusion conditions shapes the physical, functional, rheological, and antioxidant characteristics of gluten-free cereal-pulse blends, showing opportunities to tailor their properties for specific food applications; notably, formulations with a higher percentage of parboiled rice and pearl millet demonstrated greater stability and beneficial antioxidant contributions, while chickpea-high blends showed distinct emulsifying and absorption behaviors influenced by their protein and fiber content. Extrusion processing emerged as a powerful tool to modify texture and solubility, with milder conditions preserving emulsifying capacity and more intense parameters inducing marked starch and protein breakdown, thereby affecting water and oil interactions. These insights underscore the importance of selecting appropriate blend ratios and extrusion settings to develop versatile, nutrient-dense ingredients that can enhance the quality and functionality of diverse products such as soups, sauces, instant foods, and bakery goods, advancing sustainable options with health-promoting potential.

## Figures and Tables

**Table 1:** Proximate composition of the raw formulation flours.

Formulations (PR/PM/CP/CB)	Ash (g/100 g)	Protein (g/100 g)	Lipid (g/100 g)	Dietary Fiber (g/100 g)	Carbohydrates (g/100 g)
F1 (60/15/15/10)	2.19 ± 0.00 ^c^	11.20 ± 0.16 ^d^	4.41 ± 0.15 ^b^	8.81 ± 0.16 ^c^	72.40 ± 0.33 ^a^
F2 (15/60/15/10)	2.38 ± 0.05 ^cb^	12.77 ± 0.00 ^c^	5.52 ± 0.19 ^a^	11.23 ± 0.72 ^b^	66.86 ± 0.30 ^b^
F3 (15/15/60/10)	3.33 ± 0.04 ^a^	17.35 ± 0.08 ^a^	6.22 ± 0.08 ^a^	13.60 ± 0.19 ^a^	58.08 ± 0.02 ^c^
F4 (30/30/30/10)	2.56 ± 0.13 ^b^	13.64 ± 0.16 ^b^	5.62 ± 0.32 ^a^	11.10 ± 0.43 ^b^	65.93 ± 0.13 ^b^

Mean ± SD. For raw formulations, distinct lowercase letters in the same column indicate differences (*p* ≤ 0.05) among blend formulations. F1–F4 (%/%/%/%): represent blends of varying percentages of parboiled brown rice (PR), pearl millet (PM), chickpea (CP), and carioca bean (CB).

**Table 2:** Specific mechanical energy, bulk density, and particle size distribution of raw and extruded blend flours.

Blends (PR/PM/CP/CB)	Condition	Specific Mechanical Energy (SME—W·h/kg)	Bulk Density (BD—g/cm^3^)	10% Percentile D_10_ (µm)	50% Percentile D_50_ (µm)	90% Percentile D_90_ (µm)	Span (µm)
F1 (60/15/15/10)	Raw	-	^a^ 0.53 ± 0.01	^a^ 30.40 ± 0.32	^b^ 204.70 ± 6.02	^b^ 530.07 ± 12.41	^c^ 2.44 ± 0.02
F2 (15/60/15/10)	Raw	-	^b^ 0.49 ± 0.01	^b^ 26.09 ± 0.35	^c^ 174.17 ± 0.81	^c^ 496.33 ± 02.90	^b^ 2.70 ± 0.01
F3 (15/15/60/10)	Raw	-	^c^ 0.39 ± 0.01	^c^ 19.22 ± 0.27	^d^ 64.21 ± 0.66	^d^ 259.00 ± 04.20	^a^ 3.73 ± 0.04
F4 (30/30/30/10)	Raw	-	^b^ 0.49 ± 0.01	^b^ 26.65 ± 0.52	^a^ 249.27 ± 6.41	^a^ 656.93 ± 14.03	^cb^ 2.53 ± 0.12
Extruded flours							
F1 (60/15/15/10)	E1 (30/300)	073.37 ± 00.66 ^aB^	0.59 ± 0.02 ^aA^	33.50 ± 2.35 ^aA^	139.47 ± 5.76 ^aA^	287.03 ± 06.57 ^aA^	1.82 ± 0.05 ^bA^
F2 (15/60/15/10)	E1 (30/300)	058.04 ± 10.77 ^aB^	0.55 ± 0.02 ^bA^	27.22 ± 0.13 ^bA^	110.27 ± 0.59 ^bA^	233.28 ± 01.27 ^bA^	1.87 ± 0.01 ^bA^
F3 (15/15/60/10)	E1 (30/300)	056.81 ± 04.98 ^aB^	0.55 ± 0.01 ^bA^	22.32 ± 0.70 ^cB^	118.27 ± 2.83 ^bB^	272.13 ± 01.08 ^aB^	2.11 ± 0.05 ^aA^
F4 (30/30/30/10)	E1 (30/300)	066.53 ± 01.24 ^aB^	0.59 ± 0.00 ^aA^	27.04 ± 0.99 ^bB^	106.83 ± 3.35 ^bB^	229.02 ± 04.77 ^bB^	1.89 ± 0.02 ^bA^
F1 (60/15/15/10)	E2 (18/600)	208.60 ± 15.32 ^aA^	0.58 ± 0.01 ^aA^	33.22 ± 4.57 ^bA^	120.47 ± 7.09 ^cB^	234.10 ± 06.74 ^cB^	1.67 ± 0.08 ^bB^
F2 (15/60/15/10)	E2 (18/600)	185.45 ± 31.32 ^abA^	0.55 ± 0.01 ^aA^	27.47 ± 5.57 ^cA^	109.80 ± 11.07 ^cA^	240.17 ± 05.22 ^cA^	1.94 ± 0.17 ^aA^
F3 (15/15/60/10)	E2 (18/600)	151.64 ± 14.17 ^bA^	0.51 ± 0.02 ^bB^	28.08 ± 3.15 ^cA^	145.42 ± 20.53 ^bA^	303.18 ± 32.20 ^bA^	1.90 ± 0.07 ^aB^
F4 (30/30/30/10)	E2 (18/600)	171.72 ± 09.22 ^abA^	0.57 ± 0.02 ^aA^	48.63 ± 4.41 ^aA^	182.92 ± 1.50 ^aA^	329.25 ± 10.33 ^aA^	1.53 ± 0.08 ^cB^

Mean ± SD. For raw conditions, different lowercase letters at the left side of the numbers in the same column indicate differences (*p* ≤ 0.05) among blend formulations. For extruded samples, different lowercase letters at the right side of the number in the same column indicate differences (*p* ≤ 0.05) among the formulations inside the same extrusion condition. The same uppercase letters on the right side of the number in the same column indicate similarities (*p* > 0.05) between extrusion conditions for each blend formulation. E1 and E2 represent extrusion conditions (%moisture/rpm); F1–F4 (%/%/%/%): refer to blends of varying percentages of parboiled brown rice (PR), pearl millet (PM), chickpea (CP), and carioca beans (CB).

**Table 3:** Functional properties of the raw and extruded blended whole meal flour.

Blends (PR/PM/CP/CB)	Condition	WAI	WSI	OAI	EC	ES
F1 (60/15/15/10)	Raw	^a^ 2.09 ± 0.19	^d^ 7.76 ± 0.28	^b^ 1.43 ± 0.12	^c^ 9.97 ± 2.32	^b^ 68.60 ± 3.50
F2 (15/60/15/10)	Raw	^b^ 1.62 ± 0.06	^c^ 8.37 ± 0.03	^b^ 1.31 ± 0.02	^c^ b10.50 ± 1.91	^a^ 81.05 ± 0.24
F3 (15/15/60/10)	Raw	^c^ 1.28 ± 0.10	^a^ 16.82 ± 0.23	^a^ 1.72 ± 0.01	^b^ 10.71 ± 0.53	^a^ 79.60 ± 2.47
F4 (30/30/30/10)	Raw	^b^ 1.79 ± 0.05	^b^ 10.57 ± 0.04	^ba^ 1.46 ± 0.02	^a^ 19.67 ± 2.08	^c^ 52.22 ± 0.96
Extruded flours						
F1 (60/15/15/10)	E1 (30/300)	5.39 ± 0.52 ^aA^	09.64 ± 0.30 ^cbB^	0.93 ± 0.03 ^aB^	17.93 ± 1.17 ^aA^	51.11 ± 3.48 ^cA^
F2 (15/60/15/10)	E1 (30/300)	4.72 ± 0.56 ^bA^	06.78 ± 2.20 ^cB^	0.91 ± 0.02 ^abA^	15.36 ± 1.36 ^bB^	76.72 ± 2.41 ^aA^
F3 (15/15/60/10)	E1 (30/300)	3.85 ± 0.62 ^cA^	12.50 ± 1.67 ^aB^	0.89 ± 0.03 ^abB^	15.8 ± 2.82 ^bA^	56.58 ± 0.79 ^bB^
F4 (30/30/30/10)	E1 (30/300)	3.97 ± 0.13 ^cA^	10.19 ± 0.60 ^abB^	0.88 ± 0.01 ^bA^	17.9 ± 3.16 ^aA^	55.69 ± 3.36 ^bA^
F1 (60/15/15/10)	E2 (18/600)	4.26 ± 0.08 ^aB^	25.94 ± 0.59 ^aA^	1.04 ± 0.06 ^aA^	17.32 ± 0.09 ^aA^	43.15 ± 1.98 ^dB^
F2 (15/60/15/10)	E2 (18/600)	4.15 ± 0.37 ^aB^	17.84 ± 3.50 ^cA^	0.93 ± 0.03 ^bA^	16.23 ± 2.26 ^bA^	48.34 ± 1.51 ^cB^
F3 (15/15/60/10)	E2 (18/600)	2.92 ± 0.18 ^bB^	18.01 ± 2.06 ^cA^	0.93 ± 0.02 ^bA^	08.35 ± 4.55 ^dB^	87.06 ± 0.11 ^aA^
F4 (30/30/30/10)	E2 (18/600)	3.92 ± 0.12 ^aA^	21.92 ± 1.55 ^bA^	0.89 ± 0.01 ^bA^	13.64 ± 3.12 ^cB^	56.30 ± 5.65 ^bA^

Mean ± SD. For raw conditions, different lower-case letters at the left side of the numbers in the same column indicate differences (*p* ≤ 0.05) among blend formulations. For extrusion samples, different lowercase letters at the right side of the number in the same column indicate differences (*p* ≤ 0.05) among the formulations inside the same extrusion condition. The same uppercase letters on the right side of the number in the same column indicate similarities (*p* > 0.05) between extrusion conditions for each blend formulation. E1, E2 are extrusion conditions (%moisture/rpm); F1–F4 (%/%/%/%): refer to blends of varying percentages of parboiled brown rice (PR), pearl millet (PM), chickpea (CP), and carioca beans (CB).

**Table 4:** Color parameters (CIE L* a* b*) of raw and extruded flours.

Blends (PR/PM/CP/CB)	Condition	L*	a*	b*
F1 (60/15/15/10)	Raw	^b^ 82.20 ± 0.30	^c^ 0.57 ± 0.02	^b^ 15.32 ± 0.45
F2 (15/60/15/10)	Raw	^c^ 77.29 ± 0.48	^a^ 1.06 ± 0.00	^b^ 14.87 ± 0.88
F3 (15/15/60/10)	Raw	^a^ 86.05 ± 0.38	^d^ 0.05 ± 0.02	^a^ 17.05 ± 0.24
F4 (30/30/30/10)	Raw	^b^ 81.70 ± 0.28	^b^ 0.90 ± 0.03	^b^ 15.57 ± 0.22
Extruded flours				
F1 (60/15/15/10)	E1 (30/300)	82.70 ± 0.74 ^bA^	0.77 ± 0.08 ^bB^	16.46 ± 0.67 ^dB^
F2 (15/60/15/10)	E1 (30/300)	76.85 ± 0.42 ^dA^	1.19 ± 0.04 ^aB^	17.44 ± 0.22 ^cB^
F3 (15/15/60/10)	E1 (30/300)	83.41 ± 0.44 ^aA^	0.19 ± 0.05 ^cB^	20.09 ± 0.36 ^aA^
F4 (30/30/30/10)	E1 (30/300)	80.00 ± 0.49 ^cA^	0.85 ± 0.03 ^bB^	18.66 ± 0.21 ^bA^
F1 (60/15/15/10)	E2 (18/600)	82.01 ± 0.28 ^bB^	1.48 ± 0.05 ^bA^	17.93 ± 0.33 ^cA^
F2 (15/60/15/10)	E2 (18/600)	76.55 ± 0.44 ^dA^	1.80 ± 0.07 ^aA^	18.43 ± 0.26 ^bcA^
F3 (15/15/60/10)	E2 (18/600)	83.01 ± 0.32 ^aA^	0.53 ± 0.02 ^dA^	19.71 ± 0.41 ^aA^
F4 (30/30/30/10)	E2 (18/600)	80.44 ± 0.51 ^cA^	1.38 ± 0.11 ^cA^	18.67 ± 0.26 ^bA^

Mean ± SD. For raw conditions, different lowercase letters on the left side of the numbers in the same column indicate differences (*p* ≤ 0.05) among blend formulations. For extrusion samples, different lowercase letters at the right side of the number in the same column indicate differences (*p* ≤ 0.05) among the formulations inside the same extrusion condition. The same uppercase letters on the right side of the number in the same column indicate similarities (*p* > 0.05) between extrusion conditions for each blend formulation. E1 and E2 are extrusion conditions (%moisture/rpm); F1–F4 (%/%/%/%): denote blends of parboiled brown rice (PR), pearl millet (PM), chickpea (CP), and carioca beans (CB) in varying percentages.

**Table 5:** Total phenolic compound (TPC) and antioxidant capacity (ABTS^+^ and DPPH) of raw and extruded whole cereals and pulses flour blends.

Blends (PR/PM/CP/CB)	Condition	TPC	ABTS^+^	DPPH
PR	Raw	^d^ 19.37 ± 1.01	^d^ 1.86 ± 0.10	-
PM	Raw	^b^ 296.42 ± 4.64	^b^ 6.98 ± 0.15	-
CP	Raw	^c^ 60.25 ± 1.78	^c^ 3.06 ± 0.15	-
CB	Raw	^a^ 508.35 ± 7.59	^a^ 18.43 ± 0.14	-
F1 (60/15/15/10)	Raw	102.53 ± 1.52 ^c^	4.39 ± 0.39 ^c^	-
F2 (15/60/15/10)	Raw	284.91 ± 20.22 ^a^	13.20 ± 1.00 ^a^	9.94 ± 0.75 ^a^
F3 (15/15/60/10)	Raw	139.85 ± 6.59 ^b^	4.19 ± 0.05 ^c^	-
F4 (30/30/30/10)	Raw	134.00 ± 2.11 ^b^	5.51 ± 0.09 ^b^	-
F2 (15/60/15/10)	E1 (30/300)	273.00 ± 11.11 ^A^	11.62 ± 0.60 ^A^	5.76 ± 0.44 ^A^
F2 (15/60/15/10)	E2 (18/600)	238.31 ± 15.23 ^B^	10.92 ± 0.23 ^B^	5.29 ± 0.68 ^A^

Mean ± SD. For raw materials, different lowercase letters at the left side of the numbers in the same column indicate differences (*p* ≤ 0.05) among each grain. For raw conditions, different lowercase letters on the right side of the numbers in the same column indicate differences (*p* ≤ 0.05) among blend formulations. For extrusion samples, different uppercase letters at the right side of the number in the same column indicate differences (*p* ≤ 0.05) between extrusion conditions for each blend formulation. E1 and E2 are extrusion conditions (%moisture/rpm); F1–F4 (%/%/%/%): denote blends of parboiled brown rice (PR), pearl millet (PM), chickpea (CP), and carioca beans (CB) in respective proportions (%).

## Data Availability

Data will be made available on request.

## Supplementary Materials

The following supporting information can be downloaded at: https://www.mdpi.com/article/10.3390/foods14203515/s1, Table S1: Bulk density, hydration properties and oil absorption index of the non-extruded and extruded blended whole meal flour, Table S2: Particle-size distribution of the non-extruded and extruded blended whole meal flour, Table S3: Paste properties of blended Whole meal flours non-extruded and extruded running in two extrusion conditions, Figure S1: Volume percent of curves of Particle-size distribution of the non-extruded (a) and the extruded (b) blended whole meal flour.

## Abbreviations

The following abbreviations are used in this manuscript:

PM	Pearl millet
PR	Parboiled brown rice
CP	Chickpea
CB	Carioca bean
BT	Barrel temperature
SS	Screw speed
FM	Feed moisture
BD	Bulk density
PSD	Particle-size distribution
WAI	Water absorption index
WSI	Water solubility index
OAI	Oil absorption index
RVA	Rapid Visco Analyzer
PT	Pasting temperature
CV	Cold viscosity
PV	Peak viscosity
HS	Holding strength
BV	Breakdown viscosity
FV	Final Viscosity
SV	Setback viscosity
cP	Centipoise
TPC	Total phenolic compounds
EC	Emulsifying capacity
ES	Emulsifying stability
