# Supplementary material for: Impact of Whole Cereal–Pulse Flours on the Functionality and Antioxidant Properties of Gluten-Free Extruded Flours
Source: Foods. 2025 Oct 15;14(20):3515. doi: 10.3390/foods14203515 (PMC12564843; doi:10.3390/foods14203515)
Supplement: Supplementary file 1 [file foods-14-03515-s001.zip › foods-3892088-supplementary.pdf]

Impact of whole cereal-pulse flours on the functionality and antioxidant properties of gluten-free extruded flours

(Supplementary material)

Franz J. Chuqui-Paulino [1], Davy W. H. Chávez [1], José L. Ramírez Ascheri [2], Caroline Mellinger-Silva [2], Jhony W. Vargas-Solorzano [2], and Carlos W. Piler Carvalho [12*]

[1] Post graduate Program in Food Science and Technology at Federal Rural University of Rio de Janeiro, 23890-000, Seropédica, Rio de Janeiro, Brazil
[2] Embrapa Agroindústria de Alimentos, Avenida das Américas 29501, Guaratiba, 23020-470 Rio de Janeiro, Brazil.
* Correspondence: carlos.piler@embrapa.br

Table S1. Bulk density, hydration properties and oil absorption index of the non-extruded and extruded blended whole meal flour.

Sample	Condition	BD	WAI	WSI	OAI
		Non-extruded samples			
F1	NE	0.53 ± 0.01^{a}	2.09 ± 0.19^{a}	7.76 ± 0.28^{d}	1.43 ± 0.12^{b}
F2	NE	0.49 ± 0.01^{b}	1.62 ± 0.06^{b}	8.37 ± 0.03^{c}	1.31 ± 0.02^{b}
F3	NE	0.39 ± 0.01^{c}	1.28 ± 0.10^{c}	16.82 ± 0.23^{a}	1.72 ± 0.01^{a}
F4	NE	0.49 ± 0.01^{b}	1.79 ± 0.05^{b}	10.57 ± 0.04^{b}	1.46 ± 0.02^{ba}
		Extruded samples			
F1	E1	0.59 ± 0.02^{Aa}	5.39 ± 0.52^{Aa}	9.64 ± 0.30^{CBb}	0.93 ± 0.03^{Ab}
F2	E1	0.55 ± 0.02^{Ba}	4.72 ± 0.56^{Ba}	6.78 ± 2.20^{Cb}	0.91 ± 0.02^{ABa}
F3	E1	0.55 ± 0.01^{Ba}	3.85 ± 0.62^{Ca}	12.50 ± 1.67^{Ab}	0.89 ± 0.03^{ABb}
F4	E1	0.59 ± 0.00^{Aa}	3.97 ± 0.13^{Ca}	10.19 ± 0.60^{BAb}	0.88 ± 0.01^{Ba}
F1	E2	0.58 ± 0.01^{Aa}	4.26 ± 0.08^{Ab}	25.94 ± 0.59^{Aa}	1.04 ± 0.06^{Aa}
F2	E2	0.55 ± 0.01^{Aa}	4.15 ± 0.37^{Ab}	17.84 ± 3.50^{Ca}	0.93 ± 0.03^{Ba}
F3	E2	0.51 ± 0.02^{Bb}	2.92 ± 0.18^{Bb}	18.01 ± 2.06^{Ca}	0.93 ± 0.02^{Ba}
F4	E2	0.57 ± 0.02^{Aa}	3.92 ± 0.12^{Aa}	21.92 ± 1.55^{Ba}	0.89 ± 0.01^{Ba}

NE: Non-extruded; E1: first condition; E2: Second condition; F1: Parboiled rice (60), Pearl millet (15), chickpea (15), Carioca beans (10); F2: Parboiled rice (15), Pearl millet (60), chickpea (15), Carioca beans (10); F3: Parboiled rice (15), Pearl millet (15), chickpea (60), Carioca beans (10); F4: Parboiled rice (30), Pearl millet (30), chickpea (30), Carioca beans (10); BD, Bulk density; WAI, Water absorption index; WSI, water solubility OAI, index and oil absorption index.

Table S2. Particle-size distribution of the non-extruded and extruded blended whole meal flour.

Sample	Condition	D_{10}	D_{50}	D_{90}	$D_{[4,3]}$	$D_{[3,2]}$	Span
				Non-extruded			
F1	NE	30.4 ± 0.32^{a}	204.7 ± 6.02^{b}	530.07 ± 12.41^{b}	722.7 ± 7.95^{b}	606.08 ± 9.4^{b}	2.44 ± 0.02^{c}
F2	NE	26.09 ± 0.35^{b}	174.17 ± 0.81^{c}	496.33 ± 2.90^{c}	706.21 ± 10.14^{b}	585.42 ± 6.49^{b}	2.7 ± 0.01^{b}
F3	NE	19.22 ± 0.27^{c}	64.21 ± 0.66^{d}	259 ± 4.20^{d}	335.56 ± 15.96^{c}	288.72 ± 10.39^{c}	3.73 ± 0.04^{a}
F4	NE	26.65 ± 0.52^{b}	249.27 ± 6.41^{a}	656.93 ± 14.03^{a}	1289.29 ± 54.39^{a}	979.68 ± 49.75^{a}	2.53 ± 0.12^{cb}
				Extruded			
F1	E1	33.5 ± 2.35^{Aa}	139.47 ± 5.76^{Aa}	287.03 ± 6.57^{Aa}	339.73 ± 2.75^{Aa}	293.88 ± 3.98^{Aa}	1.82 ± 0.05^{Ba}
F2	E1	27.22 ± 0.13^{Ba}	110.27 ± 0.59^{Ba}	233.28 ± 1.27^{Ba}	262.62 ± 0.94^{Ba}	231.53 ± 0.93^{Ba}	1.87 ± 0.01^{Ba}
F3	E1	22.32 ± 0.7^{Cb}	118.27 ± 2.83^{Bb}	272.13 ± 1.08^{Ab}	337.5 ± 4.14^{Ab}	288.37 ± 2.44^{Ab}	2.11 ± 0.05^{Aa}
F4	E1	27.04 ± 0.99^{Bb}	106.83 ± 3.35^{Bb}	229.02 ± 4.77^{Bb}	260.66 ± 1.32^{Bb}	228.91 ± 2.65^{Bb}	1.89 ± 0.02^{Ba}
F1	E2	33.22 ± 4.57^{Ba}	120.47 ± 7.09^{Cb}	234.1 ± 6.74^{Cb}	258.91 ± 3.72^{Bb}	229.48 ± 4.25^{Bb}	1.67 ± 0.08^{Bb}
F2	E2	27.47 ± 5.57^{Ca}	109.8 ± 11.07^{Ca}	240.17 ± 5.22^{Ca}	282.01 ± 8.36^{Ba}	244.46 ± 4.00^{Ba}	1.94 ± 0.17^{Aa}
F3	E2	28.08 ± 3.15^{Ca}	145.42 ± 20.53^{Ba}	303.18 ± 32.20^{Ba}	359.83 ± 32.41^{Aa}	312.31 ± 30.07^{Aa}	1.9 ± 0.07^{Ab}
F4	E2	48.63 ± 4.41^{Aa}	182.92 ± 1.50^{Aa}	329.25 ± 10.33^{Aa}	374.63 ± 23.77^{Aa}	328.58 ± 16.95^{Aa}	1.53 ± 0.08^{Cb}

NE: Non-extruded; E1: first condition; E2: Second condition; F1: Parboiled rice (60), Pearl millet (15), chickpea (15), Carioca beans (10); F2: Parboiled rice (15), Pearl millet (60), chickpea (15), Carioca beans (10); F3: Parboiled rice (15), Pearl millet (15), chickpea (60), Carioca beans (10); F4: Parboiled rice (30), Pearl millet (30), chickpea (30), Carioca beans (10); D_{10}: small particles diameter; D_{50}: intermediate particles diameter; D_{90}: coarse particles diameter; $D_{[4,3]}$: diameter media volumetric; $D_{[3,2]}$: superficial media.

Table S3. Paste properties of blended Whole meal flours non-extruded and extruded running in two extrusion conditions.

Sample	Condition	TP	CV	PV	HS	BV	FV	SV
**Non-extruded samples**								
F1	NE	72.45 ± 1.55^{a}	32.50 ± 2.50^{b}	321.00 ± 6.00^{c}	319.50 ± 5.50^{a}	1.50 ± 0.50^{d}	843.50 ± 10.5^{b}	524.00 ± 5.00^{b}
F2	NE	73.53 ± 1.13^{a}	37.00 ± 7.00^{a}	354.50 ± 3.50^{a}	251.50 ± 2.50^{c}	103.00 ± 1.00^{c}	981.00 ± 4.00^{a}	729.50 ± 6.50^{a}
F3	NE	75.08 ± 0.42^{a}	27.00 ± 3.00^{c}	307.00 ± 2.00^{c}	280.50 ± 1.50^{b}	26.50 ± 0.50^{b}	650.00 ± 4.00^{d}	369.50 ± 2.50^{d}
F4	NE	75.50 ± 0.00^{a}	27.00 ± 3.00^{c}	244.50 ± 2.50^{b}	233.50 ± 4.50^{c}	11.00 ± 2.00^{a}	705.00 ± 5.00^{c}	471.50 ± 0.50^{c}
**Extruded samples**								
F1	E1	25.10 ± 0.00^{Aa}	506.00 ± 5.66^{Aa}	527.50 ± 9.19^{Aa}	47.00 ± 0.00^{Ba}	422.50 ± 9.19^{Aa}	427.50 ± 6.36^{Ba}	322.50 ± 6.36^{Ba}
F2	E1	25.13 ± 0.04^{Aa}	202.00 ± 5.66^{Cb}	318.00 ± 1.41^{Da}	49.00 ± 4.24^{Ca}	232.00 ± 2.83^{Cb}	279.00 ± 7.07^{BD}	193.00 ± 11.31^{Da}
F3	E1	25.10 ± 0.07^{Aa}	208.50 ± 9.19^{Cb}	419.50 ± 10.61^{Ba}	36.00 ± 7.07^{Aa}	249.50 ± 3.54^{Ca}	546.00 ± 1.41^{Aa}	376.00 ± 5.66^{Aa}
F4	E1	25.18 ± 0.04^{Aa}	273.00 ± 1.41^{Bb}	371.00 ± 2.83^{Ca}	23.50 ± 2.83^{Ca}	289.00 ± 0.00^{Ba}	309.00 ± 0.00^{Ca}	227.00 ± 2.83^{Ca}
F1	E2	25.15 ± 0.00^{Aa}	401.50 ± 6.16^{Ab}	393.50 ± 3.41^{Ab}	115.25 ± 0.00^{Ab}	346.50 ± 3.40^{Ab}	127.00 ± 1.41^{Bb}	80.00 ± 1.41^{Bb}
F2	E2	25.18 ± 0.04^{Aa}	329.50± 10.61^{Ba}	313.50 ± 2.12^{Ba}	38.00 ± 1.41^{Ab}	264.50 ± 3.54^{Ba}	102.50 ± 3.54^{Cb}	53.50 ± 2.12^{Cb}
F3	E2	25.13 ± 0.04^{Aa}	250.50 ± 7.78^{Ca}	222.00 ± 2.83^{Cb}	79.50 ± 1.41^{Bb}	186.00 ± 4.24^{Cb}	156.00 ± 0.00^{Ab}	120.00 ± 1.41^{Ab}
F4	E2	25.20 ± 0.00^{Aa}	301.50 ± 6.36^{Ba}	280.50 ± 4.95^{Bb}	29.25 ± 2.12^{Cb}	257.00 ± 2.83^{Bb}	104.50 ± 4.95^{Cb}	81.00 ± 2.83^{Bb}

NE: Non-extruded; E1: first condition; E2: Second condition; F1: Parboiled rice (60), Pearl millet (15), chickpea (15), Carioca beans (10); F2: Parboiled rice (15), Pearl millet (60), chickpea (15), Carioca beans (10); F3: Parboiled rice (15), Pearl millet (15), chickpea (60), Carioca beans (10); F4: Parboiled rice (30), Pearl millet (30), chickpea (30), Carioca beans (10); PT: Paste temperature (ºC); CV: Cold viscosity (cP); PV: peak viscosity (cP); HS: holding strength(cP); BV: breakdown viscosity(cP); FV: Final viscosity(cP); SV: Setback viscosity(cP).

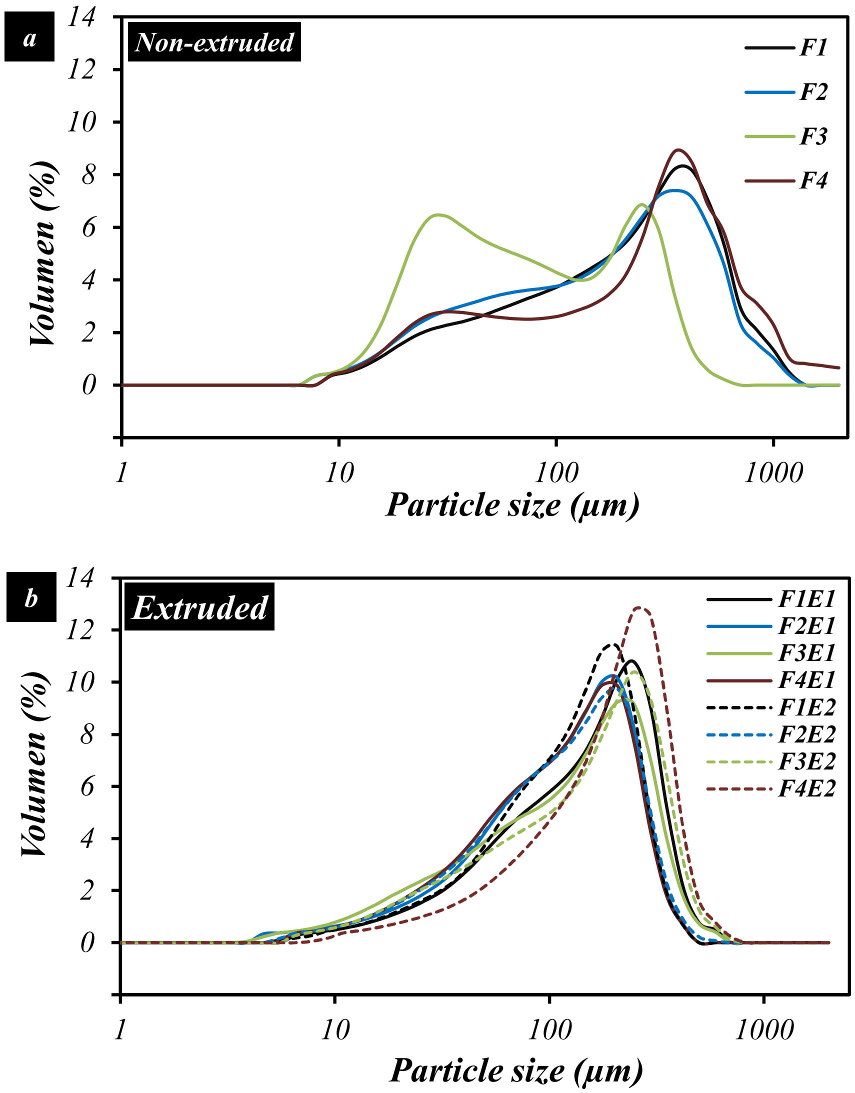

Figure S1. Volume percent of curves of Particle-size distribution of the non-extruded (a) and the extruded (b) blended whole meal flour.
